# Effect of Fucoidan Extracted from Mozuku on Experimental Cartilaginous Tissue Injury

**DOI:** 10.3390/md10112560

**Published:** 2012-11-13

**Authors:** Tomohiro Osaki, Koudai Kitahara, Yoshiharu Okamoto, Tomohiro Imagawa, Takeshi Tsuka, Yasunari Miki, Hitoshi Kawamoto, Hiroyuki Saimoto, Saburo Minami

**Affiliations:** 1 Department of Veterinary Clinical Medicine, School of Veterinary Medicine, Tottori University, 4-101 Koyama Minami, Tottori 680-8553, Japan; Email: sakurayama@ninus.ocn.ne.jp (K.K.); yokamoto@muses.tottori-u.ac.jp (Y.O.); imagawat@muses.tottori-u.ac.jp (T.I.); tsuka@muses.tottori-u.ac.jp (T.T.); minami@muses.tottori-u.ac.jp (S.M.); 2 Marine Products Kimuraya, 3307 Watari, Sakaiminato, Tottori 684-0072, Japan; Email: miki@mozuku-1ban.jp (Y.M.); kawamoto@mozuku-1ban.jp (H.K.); 3 Graduate School of Engineering, Tottori University, 4-101 Koyama Minami, Tottori 680-8553, Japan; Email: saimoto@chem.tottori-u.ac.jp

**Keywords:** articular cartilage, fucoidan, rabbit

## Abstract

We investigated the effect of fucoidan, a sulfated polysaccharide, on acceleration of healing of experimental cartilage injury in a rabbit model. An injured cartilage model was surgically created by introduction of three holes, one in the articular cartilage of the medial trochlea and two in the trochlear sulcus of the distal femur. Rabbits in three experimental groups (F groups) were orally administered fucoidan of seven different molecular weights (8, 50, 146, 239, 330, 400, or 1000 kD) for 3 weeks by screening. Control (C group) rabbits were provided water *ad libitum*. After the experimental period, macroscopic examination showed that the degree of filling in the fucoidan group was higher than that in the C group. Histologically, the holes were filled by collagen fiber and fibroblasts in the C group, and by chondroblasts and fibroblasts in the F groups. Image analysis of Alcian blue- and safranin O-stained F-group specimens showed increased production of glycosaminoglycans (GAGs) and proteoglycans (PGs), respectively. Some injured holes were well repaired both macroscopically and microscopically and were filled with cartilage tissues; cartilage matrices such as PGs and GAGs were produced in groups F 50, F 146, and F 239. Thus, fucoidan administration enhanced morphologically healing of cartilage injury.

## 1. Introduction

Our joints make complex movements easily because articular cartilage and subchondral bone absorb exercise-induced physical impact. Normal hyaline cartilage consists of chondrocytes (≤5% total volume) and extracellular matrix (≥95% total volume). Matrix consists of 70%–80% water, 20%–25% collagen, and 5%–10% proteoglycans (PGs) [1]. The collagen triple-helix consists of a repeating (Gly-X-Y)*n* sequence, and cartilage contains mixed fibrils of collagen type II, IV, IX, and XI. Proteoglycans act to pump water inside dense fiber networks and have the function of expanding pressure [2]. Aggrecan, the large species of PGs found in cartilage, has a crucial function in distributing the load in weight-bearing joints [3]. Degenerative joint disease (DJD) in humans results from excessive exercise combined with the natural aging processes [4]. 

In recent years, various combinations of non-steroid anti-inflammatory drugs, steroids, surgical treatment, oral administration of glucosamine and/or collagen supplements, weight control, and restriction of physical activity have been used for supportive treatment of patients with DJD. The glucosamine and/or collagen supplements have low toxicity and are suitable for long-term administration, although they have low analgesic effects [5,6]. In the 1980s, glucosamine (GlcN) in particular was used in the management of DJD (primarily in Europe) and had a reputation for providing clinical symptom relief [7]. We have reported accelerated healing of experimental cartilage damage by GlcN [8]. Chondroitin sulfate (CS) is also thought to heal cartilage damage, and oral CS at a dose of 800 mg per day was reported to be a pain relieving agent for the treatment of DJD. In addition, CS might stabilize the joint space width and modulate bone and joint metabolism [9]. 

Degenerative joint disease is primarily characterized by areas of destruction of articular cartilage and synovitis. Articular damage and synovitis are secondary to local increases of pro-inflammatory cytokines (interleukin-1b and tumor necrosis factor-α), enzymes with proteolytic activity (matrix metalloproteinases), and enzymes with pro-inflammatory activity (cyclooxygenase-2 and nitric oxide synthase-2). Enhanced expression of these proteins in chondrocytes and synovial membrane appears to be associated with the activation and nuclear translocation of nuclear factor-κB (NF-κB). CS reduces NF-κB nuclear translocation, probably by diminishing extracellular levels of signal-regulated kinase 1/2, p38 mitogen-activated protein kinase, and c-Jun *N*-terminal kinase activity [10]. The sulfate salt of GlcN stimulates *in vitro* production of aggrecan core protein mRNA and protein, and at the same time inhibits production and enzymatic activity of matrix-degrading MMP-3 in chondrocytes cultured from osteoarthritic articular cartilage [11]. These observations suggest that materials containing the sulfate group are prominently involved in the prevention and treatment of DJD. 

Fucoidan, which is extracted from brown algae such as kelp, wakame, and mozuku, is a sulfated polysaccharide with L-fucose building blocks and predominantly α1–2 and α1–4 linkages. Fucoidan extracted from Okinawa mozuku contains fucose (30.9%), glucose (2.2%), xylose (0.7%), uronic acid (23.4%), and sulfate groups (15.1%). Differences in composition influence the anti-inflammatory effects, anti-angiogenic effects, and anti-adhesive action [12]. Differences in the absorption and bioavailability of the various CS formulations are strongly influenced by the structure and characteristics, such as molecular mass, charge density, and clusters of disulfated disaccharides [13]. These reports suggest that differences in the molecular weight of various fucoidan preparations influence their bioactivity.

The objective of the present study was to investigate the morphological effects of orally administered fucoidans of various molecular weights on cartilage repair by screening.

## 2. Results

### 2.1. General Condition

In the C group, one rabbit presented with swelling of a stifle joint. In the F groups, 19% of the rabbits presented with swelling of stifle joints (F 8 group, *n* = 2; F 239 group, *n* = 1; F 330 group, *n* = 1). Nineteen percent of the rabbits also presented with diarrhea (F 400 group, *n* = 2; F 1000 group, *n* = 2).

### 2.2. Changes in Muscle Weight

The weights of the lateral great muscle and biceps muscle were not significantly different between the C group and the F groups. 

### 2.3. Macroscopic Findings

**Figure 1:**
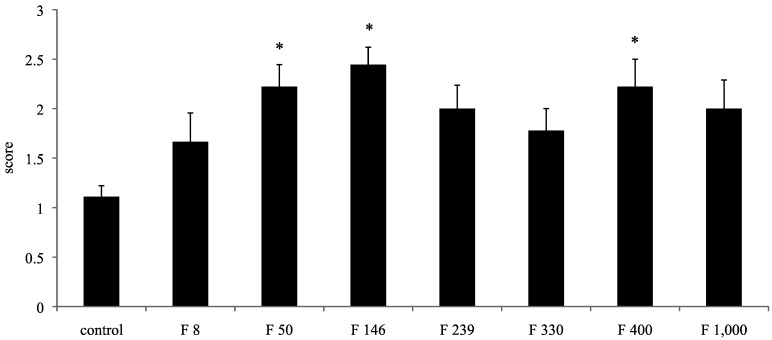
Average defect restoration scores. Data are expressed as the average ± standard deviation. * *p* < 0.05.

In the C group, the surgically created holes were incompletely healed. In the F groups, the holes were filled with regenerated tissue, though there was variability in the degree of healing. The average scores for restoration of the defective pores in the C group, F 8 group, F 50 group, F 146 group, F 239 group, F 330 group, F 400 group, and F 1000 group were 1.10, 1.67, 2.22, 2.44, 2.00, 1.78, 2.22, and 2.00, respectively. The surgically created holes in the F 50 group, F 146 group, and F 400 group exhibited significantly more healing than was found in the C group specimens (*p* < 0.05) (Figure 1).

### 2.4. Histological Findings

**Figure 2:**
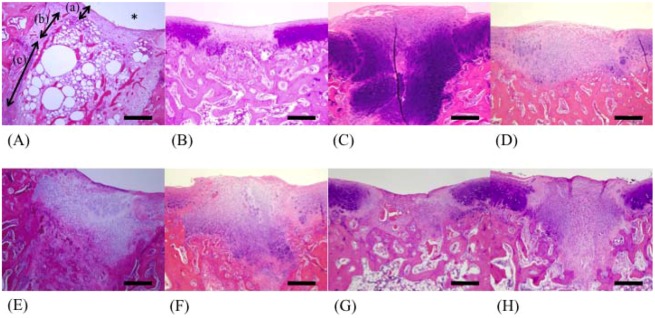
Light microscopy of tissues from each group (HE stain; 40×). (**A**) C group; (**B**) F 8 group; (**C**) F 50 group; (**D**) F 146 group; (**E**) F 239 group; (**F**) F 330 group; (**G**) F 400 group; (**H**) F 1000 group. (a) cartilage layer; (b) superficial layer of cancellous bone; (c) deeper layer of cancellous bone. * joint cavity.

In the control group, cartilage tissues were not observed in the deeper layer (Figure 2A), and bone trabeculae were lacking.

**Figure 3:**
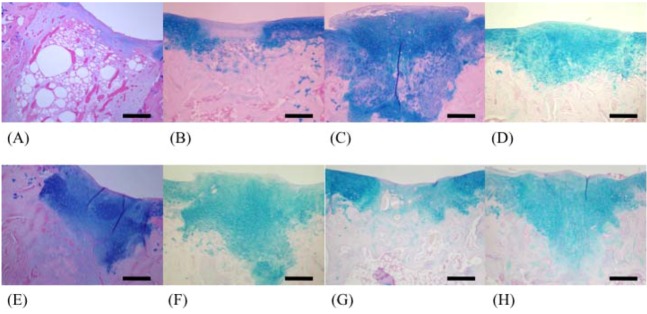
Light microscopy of tissues from each group (Alcian blue stain; ×40). (**A**) C group; (**B**) F 8 group; (**C**) F 50 group; (**D**) F 146 group; (**E**) F 239 group; (**F**) F 330 group; (**G**) F 400 group; (**H**) F 1000 group.

**Figure 4:**
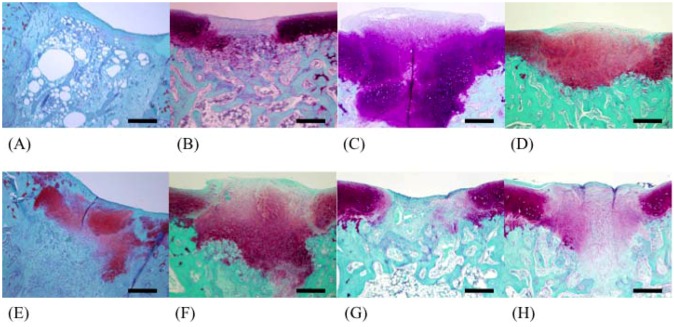
Light microscopy of tissues from each group (safranin O stain; 40×). (**A**) C group; (**B**) F 8 group; (**C**) F 50 group; (**D**) F 146 group; (**E**) F 239 group, (**F**) F 330 group; (**G**) F 400 group; (**H**) F 1000 group.

In the fucoidan group, regenerated cartilage tissues were observed in the deeper layer of cancellous bone (Figure 3F and Figure 4F), and bone trabeculae were regenerated in the F 50 and F 146 groups (Figure 4D). The greatest degree of regeneration of cartilage tissues was observed in the F 146 group. 

### 2.5. Image Analysis for Safranin O- and Alcian Blue-Stained Specimens

**Figure 5:**
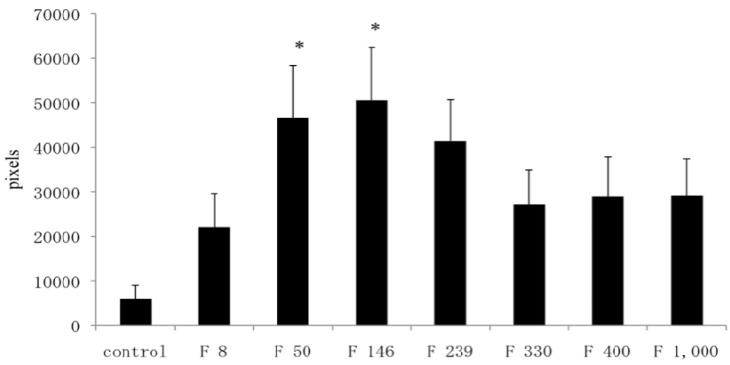
Image analysis of Alcian blue-stained specimens. Data are expressed as the average ± standard deviation. *p < 0.05.

In Alcian blue-stained specimens, the number of pixels in the C group, F8 group, F 50 group, F 146 group, F 239 group, F 330 group, F 400 group, and F 1000 group were 5989, 22,087, 46,617, 50,580, 41,392, 27,175, 28,967, and 29,146, respectively. The number of pixels in the F 50 and F 146 groups was significantly greater than that in the C group (*p* < 0.05) (Figure 5).

**Figure 6:**
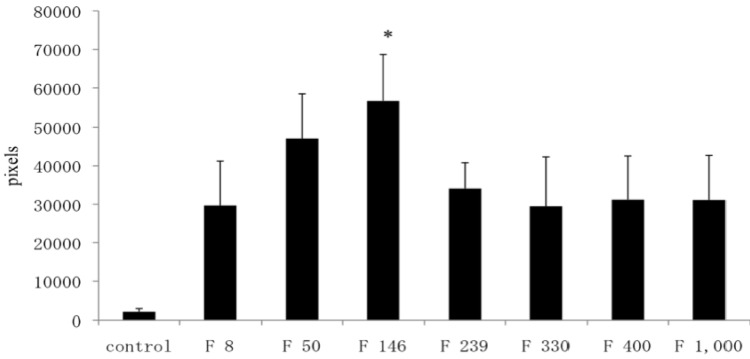
Image analysis of safranin O-stained specimens. The data are expressed as the average ± standard deviation. * *p* < 0.05.

In safranin O-stained specimens, the number of pixels in the C group, F 8 group, F 50 group, F 146 group, F 239 group, F 330 group, F 400 group, and F 1000 group were 2018, 29,686, 47,083, 56,788, 34,085, 29,474, 31,163, and 31,096, respectively. The number of pixels in the F 50 group and F 146 group were significantly higher than that in the C group (*p* < 0.05) (Figure 6). 

## 3. Discussion

Oral administration of GlcN at a dose of 1.5 g/day is effective for DJD in humans at a dose of 30 mg per kilogram of body weight [14,15]. In this study, 1 g/head of fucoidan was administered to rabbits. The dose was 500 mg/kg, which is a high dose. Nineteen rabbits developed mild diarrhea during the experiment. Although it is still unclear whether fucoidan is absorbed in the digestive tract, Becker *et al.* reported that fucoidan inhibits the absorption of trace elements [16]. In this study, diarrhea may have been caused because the high dose of fucoidan was not absorbed and was eliminated with unabsorbed foods.

Repair of cartilage injury involves fibrous tissue, fibrocartilaginous tissue, or hyaline cartilage tissue [17]. In many cases, osteochondral defects heal by formation of fibrous tissue or fibrocartilaginous tissue. Eventual deterioration of these tissues is the usual sequel to this imperfect healing. One study on cartilage injury reported that the regenerated tissue at the injured area after 12 months was fibrous rather than cartilage tissue [18]. In healed cartilage injury, the late morphological changes to a more fibrous texture might result from a loss of PGs rather than from an overt transition of all tissues to fibrocartilage [19]. In our previous study using GlcN, the holes were completely filled by proliferating chondroblasts; remodeling of bony trabeculae was also observed [20]. Therefore, our present study design was based on the period 3 weeks after surgery to enable comparison between our studies. In addition, regenerated tissue analyzed more than 3 weeks after cartilage injury has been reported to be non-functional fibrous tissue instead of hyaline cartilage [8,20]. Osteochondral defects may not heal with cartilage tissues because of the slow transition of fibrin clots into undifferentiated mesenchyme and insufficient amounts of fundamentally important regulatory factors [21].

Both chondroblasts and osteoblasts are derived from primitive mesenchymal cells of the bone marrow [22], and the undifferentiated cells that migrate to the injury site secrete primarily type I collagen, along with smaller amounts of other extracellular matrix components [17]. Furthermore, differentiated cartilage cells secrete type II collagen and PGs, and macrophages activated by these factors produce chondrocyte-stimulating factor [23]. Because direct quantification of collagen was not possible in our study, bone morphogenetic proteins (BMPs) in plasma, which are members of the TGF-β superfamily of molecules, were measured. BMPs showed a marked tendency to increase (data not shown) in the F 146.

In this study, well-repaired injuries were observed in the F 8, F 330, F 400, and F 1000 groups. However, individual variability was considerable, and many of the injured holes were filled with fibrous tissues, as in the C group. On the other hand, some injured holes were well repaired both macroscopically and microscopically and were filled with cartilage tissues; cartilage matrices such as PGs and GAGs were produced in the F 50, F 146, and F 239 groups. In a previous study using an injured cartilage model, the main collagen found in the repaired tissue after 3 weeks was type I. By 6 to 8 weeks, type II had become predominant and continued to be enriched for up to 1 year. However, type I still persisted as a significant constituent of the repair tissue even after 1 year, so the repaired cartilage never fully resembled normal articular cartilage [19]. In contrast, the present study found that the injury holes leading to cancellous bone were completely filled by proliferating chondroblasts, and remodeling of bony trabeculae was observed in the F 146 group. 

The difference between the mechanism of action of GlcN and that of fucoidans is unclear. GlcN is a monomer and is absorbed in the gut. Maximam plasma GlcN concentrations are reached at 0.5 hr after oral administration of GlcN [24]. Absorbed GlcN influences the metabolism of amino acids associated with collagen synthesis [25]. In contrast, fucoidan is a polymer composed of fucose, and an increase in plasma fucose was not observed in this study (data not shown). Because fucoidan is a sulfated polysaccharide, it was considered to be not directly associated with the synthesis of collagen. Although PGs and GAGs were evaluated in the present study, it might need to estimate the synthesis of collagen in future. We therefore suspect that the mechanism of hole repair may differ between GlcN and fucoidan. We also noted that the individual variability seen in this study was more marked than that in studies using glucosamine. Further investigation is required to evaluate the relationship between the molecular weight of fucoidans and their bioactive effects, such as collagen synthesis.

## 4. Experimental Section

### 4.1. Preparation of Fucoidan

Fucoidans with varying average molecular weight (330, 400, and 1000 KD) were extracted from Okinawa mozuku with hot water. Extracted fucoidans (molecular weight: 330 KD) were further treated under hydrothermal conditions for conversion to low-molecular weight fucoidans (8, 50, 146, and 239 KD). Fucoidans with seven different molecular weights were used for the experiment. 

### 4.2. Animals

An injured cartilage model was made in the same manner as our previous report [21]. Twenty-four clinically healthy rabbits (female Japanese albino; average age, 12 weeks) with a body weight of 2.0–2.5 kg were used. The animals were used in the experiment after 1-week acclimatization to the laboratory environment. All experimental procedures were approved by the animal care and use committees of Tottori University and were conducted in accordance with The American Physiological Society’s guiding principles for the care and use of animals.

### 4.3. Fucoidans

The experimental rabbits were divided into eight groups (*n* = 3), namely the control group (C group), the group receiving 8 KD fucoidan (F 8 group), the group receiving 50 KD fucoidan (F 50 group), the group receiving 146 KD fucoidan (F 146 group), the group receiving 239 KD fucoidan (F 239 group), the group receiving 330 KD fucoidan (F 330 group), the group receiving 400 KD fucoidan (F 400 group), and the group receiving 1000 KD fucoidan (F 1000 group). The fucoidan groups were administered a solution of fucoidan dissolved in tap water that was administered at a rate of 1 g/head/day for three weeks. 

### 4.4. Preparation of the Model of Articular Cartilage Injury

An analgesic (xylazine hydrochloride, 10 mg/kg) was administered as premedication. After sedation, induction of anesthesia was performed in a box with a mixture of 5% isoflurane in oxygen. Anesthesia was maintained by inhalation of a mixture of 3% isoflurane in oxygen using a mask. The fur at the left knee joint was clipped and the area disinfected with chlorhexidine solution (Hibiscrub, Zeneka, Osaka) and 70% alcohol. Approaching from the lateral portion of the knee joint, an incision was made vertically on the skin from the central part of the femur toward the tibial tuberosity. The articular capsule was incised, and the patella of the stifle joint was exposed completely by artificially dislocating the patella toward the medial side. Three holes measuring 2 mm in diameter and 4 mm in depth were made using a hand drill (Micro-engine D-2, Osada medical, Tokyo) at the articular cartilage of the medial trochlea (one hole) and the trochlear sulcus (two holes) of the distal femur. Afterwards, the wound was rinsed with saline solution, and the articular capsule was sutured and closed with a synthetic absorbent thread (3-0 PDSII, Johnson & Johnson, Tokyo). The subcutaneous tissues and skin were sutured with nylon (USP 3-0 suture, Suprylon, Vomel, Germany). During the 1-week period after the operation, the wound surface was disinfected by povidone-iodine (Isodine, Meiji confectionery, Tokyo) once a day, and 10 mg/kg of oxytetracycline (Terramycin, Pfizer, Tokyo) was subcutaneously administered twice a day to prevent infection.

### 4.5. Postmortem Examination

At three weeks after the operation, the rabbits were euthanized by overdose (80 mg/kg) of pentobarbital (Nembutal, Dainippon Pharmaceutical Co., Osaka) through intravenous injection. The stifle joints were opened and were macroscopically observed at the operated site for assessment of injured cartilage. The lateral great muscle and the biceps muscle in the left and right hind legs were then collected for weight comparison. The muscle weight ratio (%) was calculated by comparing the operation side with the non-operation side.

### 4.6. Evaluation of Healing at Injured Sites

For the macroscopic findings, the proportion of restoration of the defective pores was scored according to previous our report [20]: less than 50% repair of defect depth, score 0; less than 60% repair of defect depth, score 1; less than 80% repair of defect depth, score 2; and more than 80% repair of defect depth, score 3.

### 4.7. Histological Examination

The recovered left femur was fixed in a 10% neutral buffered formaldehyde solution. After fixation, the operated stifle joint was trimmed to a thickness of 5 mm and decalcified for 1 day by shaking in 5% formic acid solution. After decalcification, the tissue was soaked for neutralization in a 5% sodium sulfate solution for 1 day, and was then washed for approximately 10 h under running water. After using the usual method of paraffin embedding, the tissue was sliced by a microtome into 5-μm sections. Staining was carried out using the hematoxylin/eosin double staining method; safranin O and Alcian blue stains were used for staining PGs and GAGs, respectively. We recorded the difference between restored substances at the injured parts in all groups using a microscope (BX51-FL, Olympus, Tokyo). The 200× magnified images of restored parts, articular cartilage, and growing zone stained with safranin stains and Alcian blue stains were captured by a computer using Photograb ab-300 version 1.0 (Macintosh software, Fujifilm, Tokyo), and the images were digitized using Adobe Photoshop 3.0 (Macintosh software, Adobe System, Tokyo). In safranin stains, the red colored pixels that indicated PGs were counted in order not to include non-specific colored pixels. In Alcian blue stains, the indigo colored pixels that indicated GAGs were also counted. The proportion of each colored pixels out of a total of 120,000 pixels accounted for the observed hue (random sampling of 20,000 pixels at 6 locations) was then calculated through the image processing technique.

### 4.8. Statistical Analysis

Statistical analysis was performed using the Turkey-Kramer method. A *p* value of less than 0.05 was considered significant.

## 5. Conclusion

The administration of fucoidan effectively promoted the healing and restoration of cartilage injury. The degree of healing promoted by fucoidan may be associated with the steric structure and composition of the fucoidan as well as the size and molecular weight. Further investigations are necessary to evaluate the relationship between the molecular weight of fucoidans and their bioactive effects, such as collagen synthesis. 

## References

[1] Maroudas A., Bayliss M.T., Venn M.F. (1980). Further studies on the composition of human femoral head cartilage. Ann. Rheum. Dis..

[2] Heinegard D. (2009). Proteoglycans and more from molecules to biology. Int. J. Exp. Pathol..

[3] Hardingham T.E., Fosang A.J. (1992). Proteoglycans: Many forms and many functions. FASEB J..

[4] Barclay T.S., Tsourounis C., McCart G.M. (1998). Glucosamine. Ann. Pharmacother..

[5] Qiu G.X., Gao S.N., Giacolli G., Rovati L., Setnikar I. (1998). Efficacy and safety of glucosamine sulfate *versus* ibuprofen in patients with knee osteoarthritis. Arzneimittelforschung.

[6] Setnikar I., Giacchti C., Zanolo G. (1991). Pharmacokinetics of glucosamine in the dog and in man. Arzneimittelforschung.

[7] Deal C.L., Moskowitz R.W. (1999). Nutraceuticals as therapeutic agents in osteoarthritis: The role of glucosamine, chondroitin sulfate, and collagen hydrolysate. Rheum. Dis. Clin. North. Am..

[8] Tamai Y, Miyatake K., Okamoto Y., Takamori Y., Sakamoto K., Minami S. (2003). Enhanced healing of cartilaginous injuries by *N*-acetyl-D-glucosamine and blucuronic acid. Carbohydr. Polym..

[9] Uebelhart D., Thonar E.J., Delmas P.D., Chantraine A., Vignon E. (1998). Effects of oral chondroitin sulfate on the progression of knee osteoarthritis. Osteoarthr. Cartil..

[10] Iovu M., Dumais G., Souich P.D. (2008). Anti-inflammatory activity of chondroitin sulfate. Osteoarthr. Cartil..

[11] Dodge G.R., Jimenez S.A. (2003). Glucosamine sulfate modulates the levels of aggrecan and matrix metalloproteinase-3 synthesized by cultured human osteoarthritis articular chondrocytes. Osteoarthr. Cartil..

[12] Cumashi A., Ushakova A.N., Preobrazhenskaya E.M., Incecco D.A., Piccoli A., Totani L., Tinari N., Morozevich E.G., Berman E.A., Bilan I.M. (2007). A comparative study of the anti-inflammatory, anticoagulant, antiangiogenic, and antiadhesive activities of nine different fucoidans from brown seaweeds. Glycobiology.

[13] Volpi N. (2003). Oral absorption and bioavailability of ichthyic origin chondroitin sulfate in healthy male volunteers. Osteoarthr. Cartil..

[14] Dovanti A., Bignamini A.A., Rovati A.L. (1980). Therapeutic activity of oral glucosamine sulfate in osteoarthritis: A placebo-controlled double-blind investigation. Clin. Ther..

[15] Reichelt A., Förster K.K., Fischer M., Rovati L.C., Setnikar I. (1994). Efficacy and safety of intramuscular glucosamine sulfate in osteoarthritis of the knee. A randomised placebo-controlled, double-blind study. Arzneim. Forsch..

[16] Becker G., Osterloh K., Schafer S., Forth W., Paskins-Hurlburt A.J., Tanaka G., Skoryna S.C. (1981). Influence of fucoidan on the intestinal absorption of iron, cobalt, manganese and zinc in rats. Digestion.

[17] Suh J.K., Scherping S., Mardi T., Steadman J.R., Woo S.L.Y. (1995). Basic science of articular cartilage injury and repair. Oper. Tech. Sports Med..

[18] Mitchell N., Shepard N. (1976). The resurfacing of adult rabbit articular cartilage by multiple perforations through the subchondral bone. J. Bone Joint Surg. Am..

[19] Furukawa T., Eyre D.R., Kiode S., Glimcher M.J. (1980). Biochemical studies on repair cartilage resurfacing experimental defects in the rabbit knee. J. Bone Joint Surg. Am..

[20] Hashida M., Miyatake K., Okamoto Y., Fujita K., Matsumoto T., Morimatsu F., Sakamoto K., Minami S. (2003). Synergistic effects of D-glucosamine and collagen peptides on healing experimental cartilage injury. Macromol. Biosci..

[21] Metsaranta M., Kujala U.M., Pelliniemi L., Osterman H., Aho H., Vuorio E. (1996). Evidence for insufficient chondrocytic differentiation during repair of full-thickness defects of articular cartilage. Matrix Biol..

[22] Shapiro F., Koide S., Glimcher M.J. (1993). Cell origin and differentiation in the repair of full-thickness defects of articular cartilage. J. Bone Joint Surg. Am..

[23] Phadke K., Nanda S. (1983). Secretion of chondrocyte stimulating factor by macrophages as a result of activation with collagen and proteoglycans. Clin. Exp. Immunol..

[24] Azuma K., Osaki T., Tsuka T., Imagawa T., Okamoto Y., Takamori Y., Minami S. (2011). Effects of oral glucosamine hydrochloride administration on plasma free amino acid concentrations in dogs. Mar. Drugs.

[25] Osaki T., Azuma K., Kurozumi S., Takamori Y., Tsuka T., Imagawa T., Okamoto Y., Minami S. (2012). Metabolomic analyses of blood plasma after oral administration of D-glucosamine hydrochloride to dogs. Mar. Drugs.

